# Psychosocial Factors and Workers' Health and Safety

**DOI:** 10.1155/2015/628749

**Published:** 2015-11-08

**Authors:** Sergio Iavicoli, Giancarlo Cesana, Maureen Dollard, Stavroula Leka, Steven L. Sauter

**Affiliations:** ^1^Department of Occupational and Environmental Medicine Epidemiology and Hygiene, INAIL, Monte Porzio Catone, 00040 Rome, Italy; ^2^Research Center for Public Health, University of Milano-Bicocca, 20126 Milan, Italy; ^3^Asia Pacific Centre for Work Health and Safety, A World Health Organization Collaborating Centre in Occupational Health, University of South Australia, Adelaide, SA CA1-05, Australia; ^4^Centre for Organizational Health & Development, A World Health Organization Collaborating Centre in Occupational Health, University of Nottingham, Nottingham NG7 2RD, UK; ^5^Northern Kentucky University, Highland Heights, KY 41099, USA

Over the last decades significant developments in the economic, political, technological, and social landscape have contributed to changes in the nature of work and the way by which people work. Moreover, significant demographic and social changes have had an impact on working conditions contributing to the emergence of new risks for health at work. In this scenario, psychosocial risks have attracted the attention of occupational safety and health researchers, policy makers, and practitioners. Work-related psychosocial risks emerge from the design, content, and management of work as well as its social context that can have a hazardous influence on employees' health. They are considered as a contemporary challenge for health due to their close link with stress at work. There is evidence about the detrimental impact of work-related stress on workers' health and safety, particularly in relation to cardiovascular diseases and mental, musculoskeletal, and chronic degenerative disorders. Consequently, these issues are the primary focus of the current special issue.

Following a peer review process involving a broad group of international experts, out of over 60 submissions received, 17 contributions were accepted in this special issue. The papers selected represent a good collection of original research and review articles, with a wide geographical distribution. The contributions focus on the following: (a) work and psychosocial risks in the field of occupational health and safety, exploring the impact of psychosocial hazards in terms of workers' health, well-being, and performance and (b) policy as well as company level interventions. Thus, all of them provide new evidence-based insights in occupational health and well-being. A brief summary of each paper is presented below.

A review article “An Evaluation of the Policy Context on Psychosocial Risks and Mental Health in the Workplace in the European Union: Achievements, Challenges, and the Future” by S. Leka et al. offers a review of hard and soft law policies in the European Union in relation to mental health and psychosocial risks in the workplace, to identify strengths, weaknesses, and gaps to be addressed in the future. Ninety-four policies included in the review revealed several gaps, especially in relation to binding in comparison to nonbinding policies, and recommendations are offered for future actions in this area.

The paper “Burnout Is Associated with Reduced Parasympathetic Activity and Reduced HPA Axis Responsiveness, Predominantly in Males” by W. de Vente et al. shows the presence of a dysregulation of the sympathetic-vagal balance and the HPA axis in burnout, as indicated by elevated basal systolic blood pressure, reduced basal heart rate variability, and a trend for elevated cardiac output in the burnout group as compared to the healthy reference group. Gender differences in cardiovascular functioning and in cortisol reactivity to a psychosocial stressor and in basal alpha-amylase in the context of burnout were also found.

In their paper “Towards a Job Demands-Resources Health Model: Empirical Testing with Generalizable Indicators of Job Demands, Job Resources, and Comprehensive Health Outcomes” R. Brauchli et al. expand the logic of the original job demands-resources model from the original health impairment/motivational processes to simultaneously studying and improving pathogenic and salutogenic health development processes at work. The paper offers evidence on the applicability of this model in diverse economic sectors and professional groups and its usefulness for population-based public health interventions in the general working population.

A paper by D. Nella et al. entitled “Consequences of Job Insecurity on the Psychological and Physical Health of Greek Civil Servants” provided an estimation of short-term consequences of job insecurity associated with a newly introduced mobility framework in Greece in terms of anxiety, depression, and psychosomatic and musculoskeletal symptoms. Their findings showed immediate detrimental effects of job insecurity on the physical, psychological, and social functioning of employees.

A study reported by C. M. Ziebertz et al. in “The Relationship of On-Call Work with Fatigue, Work-Home Interference, and Perceived Performance Difficulties” focuses on the effects of the offsite on-call duties on employees' recovery from work. According to the effort-recovery model, a long lasting situation of incomplete recovery from load effects is critical for workers' health and well-being. Although the variation in the amount of exposure to on-call work was not systematically related to a lack of recovery from work, the experience of being on-call was related to fatigue, strain-based and time-based work home interference, and on-call performance difficulties.

L. F. Portela et al. explored the effect of perceived stress on insomnia symptoms, in a large sample of nurses, in their paper “Job Strain and Self-Reported Insomnia Symptoms among Nurses: What about the Influence of Emotional Demands and Social Support?” Given the high emotional demands of the nursing profession, which requires caring personal service, the role of social support in relation to work-related sleep disturbance was particularly confirmed for the emotional demand control model.

A challenge for nursing staff is exposure to verbal aggression. The paper “Verbal Aggression from Care Recipients as a Risk Factor among Nursing Staff: A Study on Burnout in the JD-R Model Perspective” by S. Viotti et al. is a cross-sectional study that examines the association between verbal aggression and burnout also considering the role of job content, social resources, and organizational resources in reducing the negative impact of verbal aggression. Authors found an association between verbal aggression and burnout that was facilitated by the job content level resources (e.g., job autonomy, role clarity, and skill discretion). It provides an interesting comparison between general nurses and nurses' aides highlighting the role of different resources in protecting nursing staff from the detrimental effects of verbal aggression on health.

In the paper “Models of Workplace Incivility: The Relationships to Instigated Incivility and Negative Outcomes” K. Holm et al. investigated workplace incivility as a social process. Different components of work incivility (experienced, witnessed, and instigated incivility) were examined also in relation to negative outcomes of workplace incivility. Witnessing coworker incivility emerged as the most important dimension to explain instigated incivility. Moreover, given the moderating role of support, organizational factors were identified as a key component to be included in future studies in this field.

Linking psychosocial risk exposure to productivity is important to draw the attention of managers and policy makers. A. Fattori et al. in their paper “Estimating the Impact of Workplace Bullying: Humanistic and Economic Burden among Workers with Chronic Medical Conditions” demonstrate the negative impact of workplace bullying on quality of life and productivity among workers with common and severe chronic diseases. Particularly, authors found a significant association between workplace bullying and all components of productivity loss as well as an association with worse health-related quality of life in comparison with other concurrent medical conditions.

The previously underresearched area of the link between workplace bullying and physical health problems is tacked in the paper “Workplace Bullying as a Risk Factor for Musculoskeletal Disorders: the Mediating Role of Job-Related Psychological Strain” by M. Vignoli et al. The researchers showed the mediating role of work-related strain in the relationship between bullying and musculoskeletal disorders of the low back, upper back, and neck, but not the shoulders. The strain process emerged as one of the elements to consider in understanding the detrimental effect of bullying on the victims' health, even though bullying remained a significant risk factor for musculoskeletal disorders.

A novel investigation by E. L. Bergsten et al. in the paper “Psychosocial Work Factors and Musculoskeletal Pain: a Cross-Sectional Study among Swedish Flight Baggage Handlers” offers an investigation of the relationship between psychosocial exposures and musculoskeletal health among flight baggage handlers. Findings showed an association between severity of pain and pain interfering with work and psychosocial factors at work (work organization, job content, interpersonal relationships, and leadership). Findings suggest the inclusion of the psychosocial work environment as a relevant target for interventions in this occupation.

The paper “Associations between Distal Upper Extremity Job Physical Factors and Psychosocial Measures in a Pooled Study” by M. S. Thiese et al. provides an exploratory analysis on the relationship between quantified job physical exposure and psychosocial outcomes in a large sample. Multiple associations between physical exposure and occupational and nonoccupational psychosocial factors were found after adjustment for age, body mass index, and gender. Moreover the study provides a quantification of this association including the effect on occupational injuries and illness.

The paper “The Association between Job Strain and Atrial Fibrillation: Results from the Swedish WOLF Study” by E. I. Fransson et al. provides additional knowledge about different risk factors related to work stress and atrial fibrillation (AF) through a two-time study. The association between job strain and AF was found to be time-dependent, since long-term exposure to job strain was more strongly associated with AF risk than shorter exposure.

Getting people back to work after sick leave for mental health problems is crucially important. In the paper “Prognostic Factors of Returning to Work after Sick Leave due to Work-Related Common Mental Disorders: A One- and Three-Year Follow-Up Study” B. Netterstrøm et al. assess the prognostic factors of return to work after one year and three years among workers after sick leave due to occupational stress. While the role of psychosocial factors in predicting return to work disappears over time, the severity of the disorder (full time sick leave and self-rated work ability) was found to be a crucial predictor in the long run.

Providing a new framework for evaluating organizational health interventions in their paper “The Context, Process, and Outcome Evaluation Model for Organisational Health Interventions” A. Fridrich et al. proposed the CPO model as a basis for a structured evaluation of combined occupational health interventions. Findings support the effectiveness of a CPO evaluation model as a shared mental model for the complex intervention evaluation process in the field of occupational health. The use of shared terminologies can facilitate the development of a common language for improving the comparability of evaluation study results.

An interesting preliminary study, “Effects of a Workplace Intervention Targeting Psychosocial Risk Factors on Safety and Health Outcomes” by L. B. Hammer et al., offers a first look at the effectiveness of a workplace intervention targeting work-life stress and safety-related psychosocial factors on health and safety outcomes. The study gives evidence of the need for focusing interventions on support training and team effectiveness for planning and problem solving to improve workers' health.

In the paper “Do Italian Companies Manage Work-Related Stress Effectively? A Process Evaluation in Implementing the INAIL Methodology” C. Di Tecco et al. offer a process evaluation on interventions to assess and manage risks related to work-related stress, using a methodological path proposed by INAIL. Findings highlight that key aspects of process and content may be considered as recurrent factors which might account for the differences in the results during the assessment phases and in the perception of the usefulness of the method.

*Sergio Iavicoli* *Giancarlo Cesana* *Maureen Dollard* *Stavroula Leka* *Steven L. Sauter*

